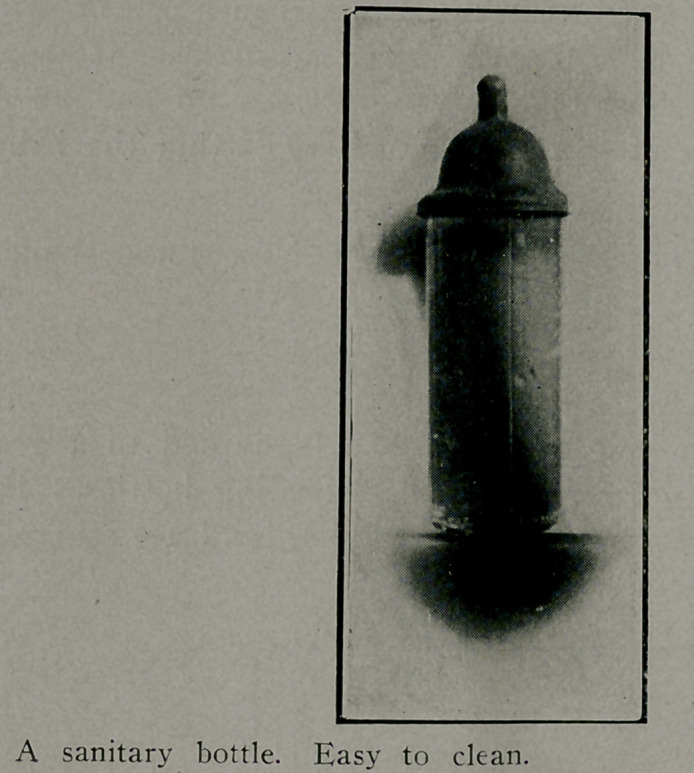# The Proper Care of Milk in the Home

**Published:** 1913-06

**Authors:** Claude A. Smith

**Affiliations:** Director Health Deparment, Laboratory of Hygiene, Atlanta, Ga.


### Journal-Record of Medicine

Successor to Atlanta Medical and Surgical Journal, Established 1855
and Southern Medical Record, Established 1870

**OWNED BY THE ATLANTA MEDICAL JOURNAL COMPANY**

PUBLISHED MONTHLY

*Official Organ Fulton County Medical Society, State Examining Board, Presbyterian Hospital, Atlanta, Birmingham and Atlantic Railroad Surgeons' Association, Chattahoochee Valley Medical and Surgical Association, Etc.*

EDGAR BALLENGER, M. D., Editor
BERNARD WOLFF, M. D., Supervising Editor
A. W. STIRLING, M. D., C. M., D. P. H.; J. S. HURT, B. Ph., M.D.
GEO. M. NILES, M. D., W. J. LOVE, M. D., (Ala.); Associate Editors
E. W. ALLEN, Business Manager

**COLLABORATORS**

DR. W. F. WESTMORLAND, General Surgery
F. W. McRAE, M. D., Abdominal Surgery
H. F. HARRIS, M. D., Pathology and Bacteriology
E. B. BLOCK, M. D., Diseases of the Nervous System
MICHAEL HOKE, M. D., Orthopedic Surgery
CYRUS W. STRICKLER, M. D., Legal Medicine and Medical Legislation
E. C. DAVIS, A. B., M. D., Obstetrics
E. G. JONES, A. B., M. D., Gynecology
R. T. DORSEY, JR., B. S., M. D., Medicine
L. M. GAINES, A. B., M. D., Internal Medicine
GEO. C. MIZELL, M. D., Diseases of the Stomach and Intestines
L. B. CLARKE, M. D., Pediatrics
EDGAR PAULIN, M. D., Opsonic Medicine
THEODORE TOEPEL, M. D., Mechano Therapy
R. R. DALY, M. D., Medical Society
A. W. STIRLING, M. D., Etc., Diseases of the Eye, Ear, Nose and Throat
BERNARD WOLFF, M. D., Diseases of the Skin
E. G. BALLENGER, M. D., Diseases of the Genito-Urinary Organs

VOLUME LX — JUNE, 1913 — No. 3

### THE PROPER CARE OF MILK IN THE HOME.

BY CLAUDE A. SMITH, M. D.,

DIRECTOR HEALTH DEPARMENT, LABORATORY OF HYGIENE, ATLANTA, GA.

Volumes have been written about the dangers of impure milk and the enormous death rate among infants and children as a result of the crimes of omission of the dairymen. Almost invariably the poor dairyman has been blamed for it all.

While the dairyman has been at fault to a certain extent, yet he is not altogether to blame, for oftentimes as much harm has resulted from improper care and handling of the milk in the home of the consumer. With the dairyman, it is generally a matter of ignorance; at heart he intends well and has no idea of the results which follow the use of milk which he has handled carelessly. He has thoughtlessly and unconsciously committed sins of omission.

Years of experience in working and studying with the city's milk supply shows that the dairyman is not the only one who is guilty of crimes of carelessness which are productive of infant mortality. Not infrequently, individuals apply at the Laboratory for protection against the dairyman whose milk has made them sick, and upon investigation we find that the consumer was at fault as much if not more than the dairyman. We find that people owning their own cows are producing sickness among their children as a result of ignorance as to the proper way of handling milk.

Nature intends that all milk should be consumed while fresh and warm, and if not so consumed, fermentation and putrefaction should follow. Milk which is fermenting or undergoing putrefaction will frequently produce poisoning, resulting in diarrheal disease,

All fermentation and putrefaction of milk is due to the action of germs which happen to fall into the milk from the air, or deposited in the milk with dust and dirt.

It is impossible to produce milk without its containing at least a small number of these germs. However, a SMALL number is not likely to produce harm in the human body. The greatest danger arises from allowing them to grow. They can not grow unless they are kept in a warm place. By a warm place, we mean a temperature ABOVE 50 DEGREES Fahrenheit. If milk is kept below 50 degrees, we find that the germs do not multiply to any extent in 24 hours, and the milk remains healthful. To protect the consumer we require the dairymen to deliver the milk at a temperature below 55 degrees. We find, however, that after the consumer receives the milk at this temperature, he does not see that it is so kept. As the temperature rises, the more the germs multiply and produce poisons. The longer time the germs have been growing the more dangerous the milk has become.

Bearing this in mind, it is perfectly plain that milk which is of an exceedingly fine quality when delivered to the consumer may become rank poison in a few hours. The object of this paper is to call to the attention of the consumer some facts which he should know in order to be sure that he is not poisoning himself by improper handling of milk.

To be fair with the dairymen, it is important that the consumer should first make a few examinations about the milk supply before placing the blame.

DAIRY THERMOMETER.

This small thermometer is necessary for proper care of milk for infant feeding. Costs but 25c.

### A Thermometer Is Indispensable.

The first requisite toward the proper care of milk in the home is a dairy thermometer: It is almost impossible to keep milk properly without this small instrument. The cost is slight (about 25 cents) and if properly used will give an almost positive check on the milk while it is in the home.

### Delivery of the Milk.

The law requires the dairymen to deliver milk at a temperature below 55 degrees. (The Health Department requested a temperature of 50 degrees, but for some unknown reason the law was placed at 55 degrees.)

Where milk is used for infant feeding, it should not be allowed to sit on the steps in the sunshine in the early morning.

The mother should know the time of the arrival of the dairymen, receive the cold milk from him, test its temperature with the thermometer, and place it next to the ice where it will keep until needed.

If the temperature at delivery is found to be 55 degrees, or above, it is her duty to notify the Health Department at once, and the matter will receive prompt attention.

Dangerous to guess at the temperature of milk. Test the milk as soon as received from the dairy.

### The Proper Refrigerator.

It is important for the consumer to know that few refrigerators, even when filled with ice, will keep a temperature which will preserve food and keep it from fermenting and spoiling. It is impossible to tell the value of the refrigerator unless it is provided with a thermometer. This same dairy thermometer will answer the purpose, and can be kept hanging in the refrigerator when not otherwise in use.

Milk "on the ice." Dangerous way to keep milk. The top milk spoils and may make the baby sick.

If the refrigerator is left to the care of the servants, it is necessary that it should be provided with a high and low registering thermometer so that the owner can tell at a glance when the doors of the refrigerator have been left open and the temperature allowed to run above the spoiling point. This high and low registering thermometer will soon pay for itself in the saving of the ice bills, and at the same time gives a check on the care of food in preserving health (which is most important of all.) The milk for the baby must be kept in a refrigerator to itself. The refrigerator should open from the top. It is dangerous to place milk in an ordinary refrigerator which has doors opening from the sides. The doors may be accidentally left slightly ajar and, if so, the milk is almost sure to spoil. As heat rises and cold descends, a refrigerator which opens from the top will not easily allow the cold to escape.

Milk "beside the ice." A better way to keep milk. The ice should always extend *above* the milk.

People frequently remark, "I place the milk directly on the ice." Now it should be borne in mind that cold goes downward, while heat risees. Therefore, the top of the refrigerator, or ice box, is always several degrees higher in temperature than the lower part. Actual tests show that the bottle of milk placed on TOP of the block of ice in a refrigerator will have a temperature of 60 degrees in the top layers of the milk while the bottom layer of the milk next to the ice will register as low as 40 degrees. If it is stirred before being tested, it will register about 50 degrees.

Therefore, a MIXTURE of the upper and bottom layers may not appear at all suspicious or dangerous. In this way the consumer may deceive himself. The top of the milk may spoil and become poisonous while the bottom layer may remain fresh. The proper way is to place the milk UNDER the ice, or beside the ice, being sure that the ice reaches a higher level than the top of the milk.

Small refrigerators, or ice boxes, especially made for milk, may be obtained from the dealers. Where it is not convenient to obtain these baby refrigerators, they can be easily made at the expense of a few cents, which will keep the milk as well at the most expensive refrigerators which open from the side. A lard tub which has been scrubbed with soap and hot water and a galvanized iron bucket placed inside, with plenty of clean excelsior packed underneath and between the iron bucket and the sides of the tube, makes an effective refrigerator. FIVE CENTS WORTH OF ICE in this will keep the milk for twenty-four hours.

### WHERE TO OBTAIN MILK.

When buying milk for infant feeding, always get it from some dairyman who comes directly from the farm. Do not purchase milk from milk depots. If possible, get milk both morning and evening. The fresher the milk the safer it is.

The City Laboratory will gladly furnish a list of dairymen delivering milk in your neighborhood with a record of each.

A number of dairies supplying milk to the City have been tested for tuberculosis, under the direction of the State Veterinarian, Dr. Bahnson, and this milk should be given preference over that which has not been tested.

In making arrangements with the dairyman, be sure to have it distinctly understood that he is to deliver you the fresh morning's milk. As people buy more milk in the morning than in the afternoon, many dairymen keep some milk over night. As this is stale milk, it should be avoided.

Do not poison the baby's milk by tasting it. Use the thermometer and be *sure* the temperature is correct.

### Preparing Milk for Infant Feeding.

It is customary for the physician to prescribe feedings for the infant for 24 hours. It is much safer to prepare these feedings every 12 hours.

When the doctor's directions require the HEATING OF FEEDINGS which are prepared for twenty-four hours, if such feedings, while still hot, are placed in the refrigerator, or ice box, it may require several hours for the bottles to become sufficiently cool to stop the growth of germs. Therefore, all the bottles of such feedings, as soon as prepared, should be cooled immediately IN ICE WATER to a temperature below 50 degrees, and then placed next to, or under the ice, in the baby's refrigerator. When these feedings are removed during the day, they should be tested with the thermometer to be sure that the ice box has preserved them properly.

### Warming the Baby s Feedings.

When it is time to feed the baby, one of the bottles should be removed from the refrigerator and placed in a pan of warm water, SHAKING THE BOTTLE EVERY FEW MINUTES. It should be heated carefully to a temperature between 95 and 98 degrees. Do not guess at the temperature by tasting the baby's milk, but USE THE THERMOMETER each time.

Milk which is too hot or too cold will upset the stomach of a young baby.

### Milk Bottles.

The health of the infant can be safeguarded to a great extent by making a proper selection of feeding bottles. Bottles which have much tubing, small opening, and are otherwise difficult to clean, should be avoided.

A bad model. Difficult to clean and therefore dangerous.

A bottle should be selected which has a large opening so as to permit easy cleansing.

All bottles should be washed immediately after being used and boiled for ten minutes before being used again.

As many children die every summer from lack of proper care in the handling of milk, the foregoing instructions cannot be followed too closely.

A sanitary bottle. Easy to clean.

The infant mortality in Atlanta is rapidly decreasing, but there is room to still further lower the death rate by the proper care and handling of milk in the home.

## Figures and Tables

**Figure f1:**
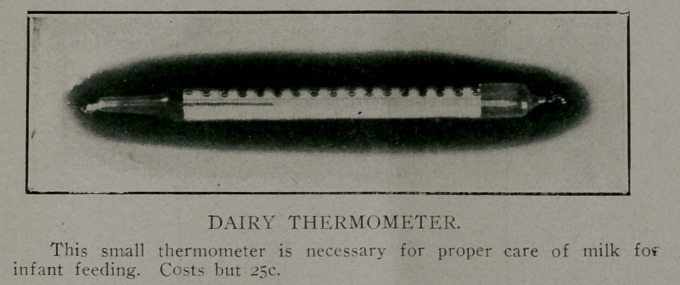


**Figure f2:**
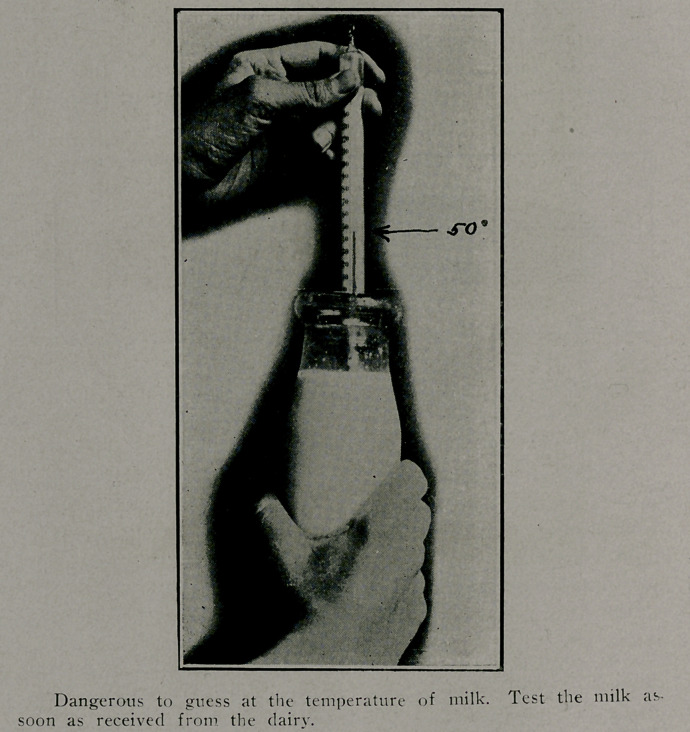


**Figure f3:**
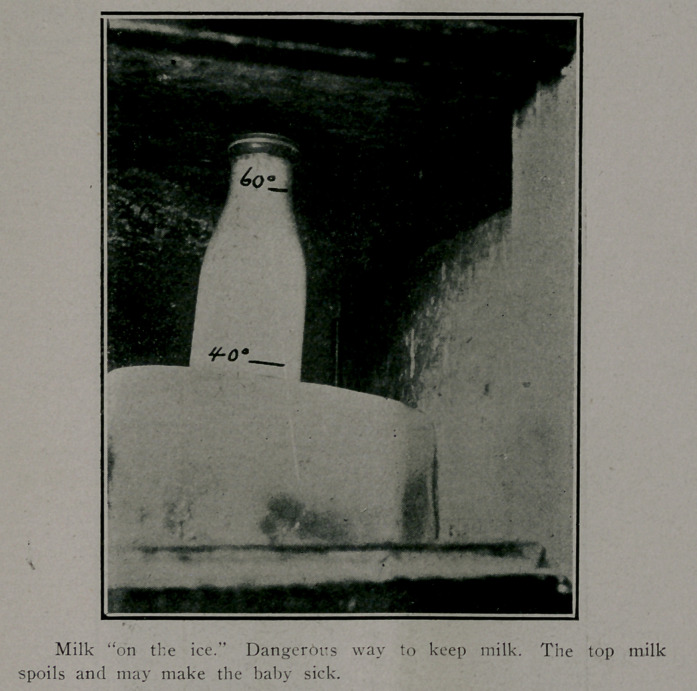


**Figure f4:**
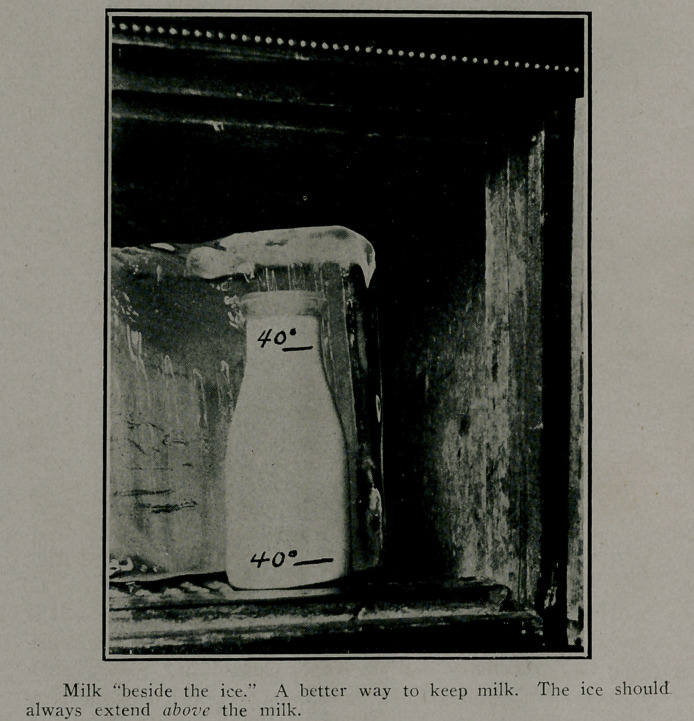


**Figure f5:**
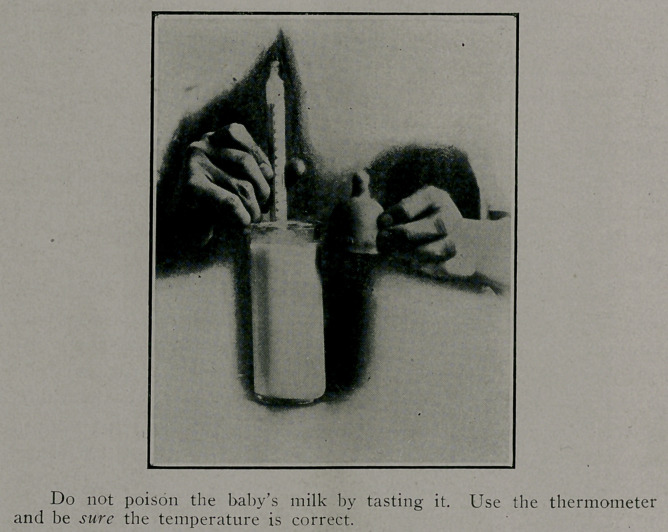


**Figure f6:**
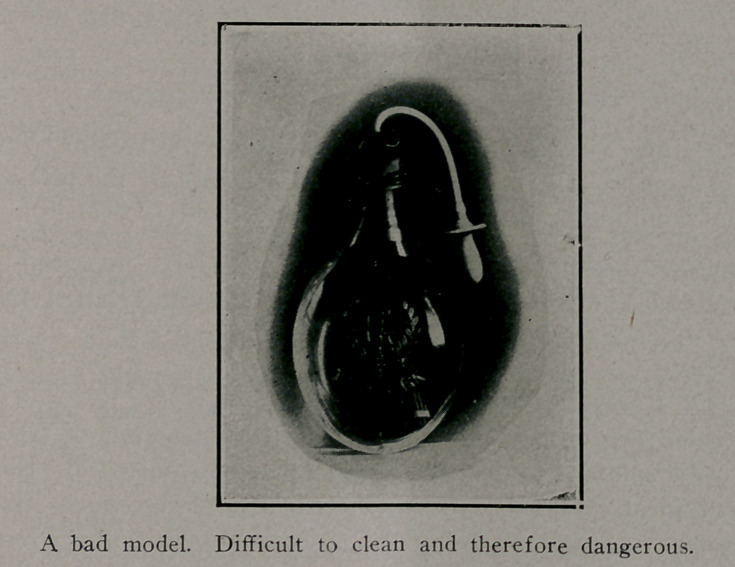


**Figure f7:**